# A Measure to Target Antipoverty Policies in the European Union Regions

**DOI:** 10.1007/s11482-014-9361-z

**Published:** 2014-09-16

**Authors:** Paola Annoni, Dorota Weziak-Bialowolska

**Affiliations:** 1 European Commission, Joint Research Centre, Modelling and Coordination of Scientific Support for Impact Assessment, Brussels, BE Belgium; 2 European Commission, Joint Research Centre, Econometrics and Applied Statistics Unit, Ispra, IT Italy

**Keywords:** EU regions, EU-SILC, FGT indices, Generalised mean, Housing costs, Multidimensional poverty

## Abstract

The reformed cohesion policy (CP), which is the major investment tool in the European Union (EU) for delivering the Europe 2020 targets, will soon make available substantial funds to improve the quality of life of the EU citizens through supporting the economic and social development of the EU’s regions and cities. Because the reformed CP has intensified the emphasis on measuring results, also with respect to reducing poverty and social exclusion, this paper is about measuring poverty to better target EU local policies. We propose a measurement of poverty at the sub-national level in the EU by means of three poverty components describing absolute poverty, relative poverty and earnings and incomes. The core data source is the cross-sectional European Statistics on Income and Living Conditions (EU-SILC) micro-data, waves 2007–2009. Data reliability at the sub-national level is statistically assessed and the regional level is described whenever possible. To calculate the poverty components, an inequality-adverse type of aggregation is applied in order to limit compensability across indicators populating a component. No aggregation is, however, performed across the three components. In the computations of income-related indicators, individual disposable income adjusted for housing costs, used as a proxy for the costs of living, is used. Poverty is confirmed to be a multi-faceted phenomenon with clear within-country variability. This variation depends on the type of region likely linked to the urbanisation level and, consequently, to the costs of living. The proposed measure may serve to better target anti-poverty measures at the local, sub-national level in the EU.

## Introduction

The European Union (EU) cohesion policy (CP) is an integrated approach to support the economic and social development of regions. One of the main objectives of the CP is to improve the level of well-being of people across the EU. The reformed CP for the period 2014–2020, approved by the European Parliament in November 2013, represents the EU’s most important investment tool for delivering the Europe 2020 targets^1^: creating growth and jobs, tackling climate change and energy dependence, and reducing poverty and social exclusion. It also sets out new conditions for funding and intensifies the emphasis on measuring results with respect to delivering the Europe 2020 targets.

Many aspects of well-being and standard of living have indeed a straightforward link to policies, which are mostly defined at regional and local levels. As such, the CP lies at the core of the EU policy objective of improving the quality of life of its citizens. However, to fulfil the objectives of the CP, it is important to know how to measure people’s quality of life. Its measurement goes well beyond Gross Domestic Product (GDP).

The rethinking of economic growth following the economic and financial crisis added another impetus to developing alternative measures of quality of life, well-being and living standards. While there are numerous initiatives and concrete examples of socioeconomic well-being indicators at the national level, the availability of regional indicators is rather scarce and usually limited to one country. There is, however, a multidimensional measure of poverty officially used in the EU, namely the ‘at risk of poverty or social exclusion’ (AROPE) rate, which is reported not only at the country level, but also for different geographical levels (NUTS levels^2^) and different density of population areas. This measure, using both income and non-income indicators and referring to the situation of people either at risk of poverty, or severely materially deprived or living in a household with a very low work intensity, informs about the shares of poor. Yet, it does not take into account other measures of poverty, like the poverty depth and intensity, and does not include the variability of the costs of living across regions. We provide a more detailed and systematic measurement of poverty in the EU regions to better target anti-poverty policies at the local level.

The paper is organised as follows. Poverty measures presents different approaches to the measurement of poverty and Conceptualisation of poverty measures describes the proposed conceptualisation of poverty. In A note on the adjustment for housing costs the importance of adjusting for costs of living is discussed, especially when going sub-national. Micro-data sources, with special emphasis on sub-national level representativeness, are presented in Data source and reliability while The three components of regional poverty presents the three poverty aggregated measures. The statistical approach, adopted for the setting-up of these aggregated measures, is presented in Aggregated measures. The distribution of poverty across EU regions presents three poverty measures and discusses our reasons for not proceeding with the computation of a final, single measure of regional poverty. Finally, Summary summarises the main outcomes.

## Poverty Measures

No one questions any longer that poverty and well-being are multidimensional concepts (Lustig 2011). Many recent studies not only address poverty by means of numerous dimensions, such as poverty in education, health and living standards, but also include monetary and non-monetary indices (Alkire and Foster 2011a, b; Antony and Visweswara Rao 2007; Atkinson et al. 2002, 2004, 2010; Betti et al. 2012; Bubbico and Dijkstra 2011; Callander et al. 2012; Merz and Rathjen 2014; Ravallion 2011; Rojas 2011; Wagle 2008; Weziak-Bialowolska and Dijkstra 2014). However, the notion of poverty is understood differently in different contexts (Callander et al. 2012). According to Wagle (2008) and Saunders (2005) there are three main approaches in the conceptualisation and operationalisation of poverty: economic well-being, capability and social inclusion. Nevertheless, an analysis of their basis and meaning reveals that the capability approach considerably stems from the economic well-being approach.

The economic well-being concept links poverty to the economic deprivation that, in turn, relates to material aspects and/or standards of living (Boulanger et al. 2009; Wagle 2008). Thus, the perfect measure of poverty in terms of economic well-being should be a combination of income, consumption and welfare. Although the measurement of income is not a problematic issue, at least to some extent, the measurement of consumption level and welfare is not straightforward. For these reasons, the level of disposable income is often used as a proxy of consumption (Decancq and Lugo 2013).

The capability approach, proposed by Sen (1993), expands the notion of poverty from welfare, consumption and income to broader concepts like freedom, well-being and capabilities. In his approach poverty is understood as a state of capability or functioning deprivation that happens when people lack freedom and opportunities to acquire or expand their abilities. Capabilities are things persons are able to do or which enable them to lead the life they currently have. Functioning represents the achievement that a person is capable of realising, or, as modified by Sen (2002) later on, the ability to make outcomes happen. Freedom is a principle determinant of individual initiative and social effectiveness that enhances the ability of individuals to help themselves, which implies that the use of freedom is part of what well-being is. According to Sen (1999) there are five distinct freedoms: political freedoms, economic facilities, social opportunities, transparency guarantees and protective security, which determine what people are ‘capable’ of becoming or doing (achieving).

The social inclusion approach is the opposite to social exclusion, which relates to a condition of systematic isolation, rejection, humiliation, lack of social support, and denial of participation (Wagle 2008). It focuses on deficiencies, while the capability approach focuses on possibilities and abilities. The last two approaches expand the economic notion of poverty by including the sociological point of view.

## Conceptualisation of Poverty Measures

In this paper we limit ourselves to poverty understood as economic well-being, or economic deprivation measured in absolute and relative terms at the sub-national level, optimally at the second level of the NUTS, namely NUTS 2, which are basic regions for the application of regional policies. It implies that no measures of poverty related to education or health, which are two of the most frequently occurring non-income poverty dimensions, are used. Although we are aware of the consequent limits, this is intentional as in this paper we focus on poverty and not on well-being or quality of life in a broader sense.

**Fig. 1:**
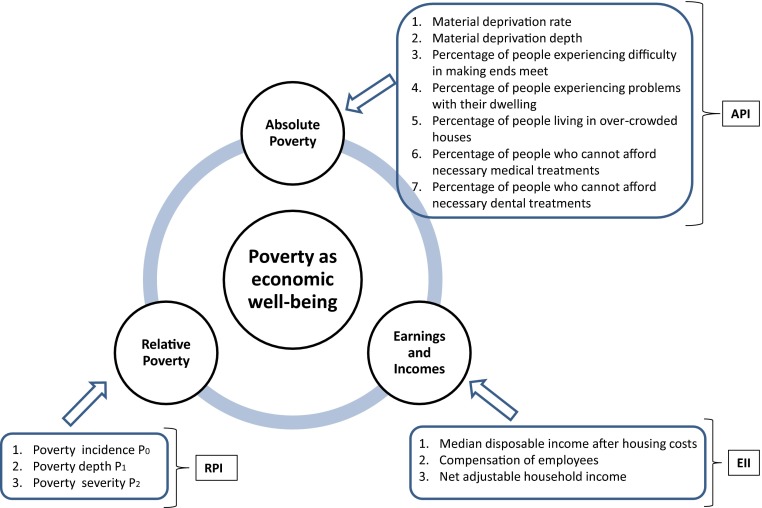
Framework of the poverty concept

The multidimensional measure of poverty at the regional level is assumed to consist of three components: 1. Absolute Poverty; 2. Relative Poverty and 3. Earnings and Incomes. Indicators populating the poverty components are listed in Fig. 1 and described in The three components of regional poverty. Their choice results from both theoretical considerations and data availability and quality.

For each component an aggregated measure, which ensures non-full compensability between indicators, is provided. It means that a deficit in one variable cannot be entirely offset by a surplus in another. To be in line with the variety of poverty definitions to assess multidimensional poverty, we use both monetary and non-monetary indicators and take into account subjective measures, by including several self-assessed indicators of absolute poverty. No direct measure of perceived poverty level is included in the analysis due to the lack of reliable data at the sub-national level.

To the best of our knowledge, our approach features the following innovative points.

i) We focus on regional variability because the EU regions, not the countries, are the key elements of the EU’s regional policy (Becker et al. 2010) and local differences in poverty are essential for targeted anti-poverty policies.
ii) We take into account the housing costs, which are a crucial factor in the computation of an individual’s disposable income as, due to highly diversified rental and purchase prices across regions, they can strongly affect actual disposable incomes.
iii) We adopt an inequality-adverse type of aggregation, generalised mean of order *β* = 0.5, within each poverty component. This approach prizes higher the improvement in those indicators that perform poorly, thus not allowing for full compensation between indicators. Such an approach is in line with recent developments in the field, such as the Human Development Index (Klugman et al. 2011), based on the geometric mean (generalised mean of order *β* = 0) since 2011 and the Material Condition Index proposed by the OECD (Ruiz 2011).

## A Note on the Adjustment for Housing Costs

The inclusion of housing costs in the computation of the individual disposable income shall be conceptually justified. Most authors do not include costs of living in their computation of disposable income and income poverty (Wagle 2008; Whiting 2004; Wong 2005). It may result from the fact that the classical definition of disposable income says that it is an income remaining after deduction of taxes and social security charges, and available to be spent or saved. However, in order to reliably compare regions, both within and across countries, the inclusion of cost of living in the estimation of actually disposable income is especially important. Indeed, as shown by Dijkstra (2013), the cost of living can differ substantially across areas with different degrees of urbanisation.

Adjusting the income for cost of living at the sub-national level is, however, quite challenging as there are no harmonised data on the within-country living costs in the EU. Still, an approximated approach can be proposed. On the one hand, it is known that services such as telecommunications, postal services and energy are provided at the same cost throughout a country and most tradable goods do not differ substantially in cost between the EU countries. On the other hand, housing costs do substantially differ between different areas of a country and between countries. Therefore, they can be seen as one of the key contributors to differences in cost of living in developed countries and thus in regional poverty distribution (Kemeny and Storper 2012; Wong 2005) 3.

It was shown by many researchers—Hutto et al. (2011), Jolliffe (2006), Kemeny and Storper (2012) and Ziliak (2010) for the United States; Van Dam et al. (2003) for Flanders; McNamara et al. (2006), Miranti et al. (2011) and Tanton et al. (2010) for Australia; and Massari et al. (2010) for Italy—that the impact of accounting for housing cost differences across rural and non-rural areas is considerable. Not adjusting for differences in cost of living leads to a significant overestimation of poverty in low -cost areas and an underestimation of poverty in high -cost areas. It may also result in a complete reversal of poverty rankings as claimed by Jolliffe (2006).

A recurrent argument against the inclusion of housing costs is that a within-country variation of housing costs is not only due to differences in prices but also due to different preferences of the households. In other words, some households are willing to pay more for housing services because they opt for different—better quality, higher size—housing solutions. This is certainly true for a number of households, but do they constitute the majority? Dijkstra (2013) recently provided the evidence of the scale of the problem by showing that:

1. housing costs in the EU cities are in all EU countries higher than the national average (the only exception is Germany);
2. housing costs in rural areas in all EU countries are lower than the national average (with the exception of Belgium).

How does this affect poverty rate related measures? In the countries with large differences in housing costs across areas with different population densities, adjusting the at-risk-of-poverty rate for housing costs will significantly change the poverty incidence. This means that housing costs do affect individual disposable income especially of the poorer part of the population, which is exactly the reason for taking them into account in a poverty-related analysis like ours.

## Data Source and Reliability

Our core data source is the cross-sectional European Statistics on Income and Living Conditions (EU-SILC) micro-data that describe different aspects of living standards at the household and individual level in the EU. Three waves (2007, 2008 and 2009) are used in the computations. In populating the poverty concepts, our requirement is to describe the sub-national level, optimally the NUTS 2 level. However, the level finally described is determined by the availability of the regional identifier in the database (Table 6. in the Appendix) and by data reliability analysis. Indicators from the Eurostat regional statistics are also used.

Being aware of country -level representativeness of the EU-SILC data, we tried to make the best use of currently available data. Methodological approaches to increase the reliability at the sub-national level of data designed to be representative at the national level only are broadly described in the literature devoted to the use of the EU-SILC (Lelkes and Zolyomi 2008; Longford et al. 2012; Verma et al. 2010; Ward 2009). The approach adopted here is rather pragmatic and combines two of the most popular approaches for the EU-SILC micro -data analysis. First, sub-national data reliability is assessed by comparing the EU-SILC weighted sample size for different gender-age classes with the Eurostat -based population share in the same gender-age classes (age classes: 0–14, 15–34, 35–54, 55–74, 75+). The level of significance of the differences is approximated by the *t*-statistic. Significant discrepancies, at level *α* = 0.05, account for 7.7, 4.0 and 0.0 % of all the cases for the EU-SILC 2007, 2008 and 2009 respectively (more details in Annoni et al. (2012)).

To reduce the impact of sample sizes detected as not reliable enough, first, the sub-national level for France is moved from NUTS 2 to NUTS 1, as also adopted by Ward (2009), who employed the EU-SILC at the sub-national level. Second, two problematic Spanish regions, namely Ciudad Autónoma de Ceuta (ES63) and Ciudad Autónoma de Melilla (ES64), are discarded from the analysis, while keeping the NUTS 2 level for the rest of the country. Then, each indicator is computed for each wave separately and then averaged across the three waves, 2007–2009 to improve the precision of the poverty measurement.^4^ Consequently, the lowest feasible and most appropriate (in terms of regional representativeness) geographical level adopted in our analysis is as follows:

- the lowest sub-national, spatial level—NUTS 2—for the Czech Republic, Spain, Finland and Romania;
- the intermediate sub-national level—NUTS 1—for Austria, Belgium, Bulgaria, France, Greece, Hungary, Italy, Poland and Sweden;
- the country level—NUTS 0—for Cyprus, Germany, Denmark, Estonia, Ireland, Lithuania, Luxembourg, Latvia, Malta, the Netherlands, Portugal, Slovenia, Slovakia and the United Kingdom.^5^

Unfortunately, many big countries lack the regional identifier in the EU-SILC database. We tried to solve this problem by examining country-specific household surveys with regard to their subnational representativeness and similarity of poverty-related questions.^6^ However, the analysis showed a non-optimal data reliability at the regional level and many comparability problems with the EU-SILC derived indicators, especially with respect to the definition of disposable income components. Results are not shown here but can be found in Annoni et al. (2012).

## The Three Components of Regional Poverty

### Absolute Poverty

The Absolute Poverty component measures the individual’s capacity to afford basic needs and includes the following indicators calculated at the regional level: (1) material deprivation rate as used by Eurostat (Törmälehto and Sauli 2010), (2) material deprivation depth, (3) percentage of people experiencing difficulty in making ends meet, (4) percentage of people experiencing problems with their dwelling, (5) percentage of people living in over-crowded houses, (6) percentage of people who cannot afford necessary medical treatments, and (7) percentage of people who cannot afford necessary dental treatments. A detailed description of the indicators is presented in Table 1.

**Table 1:** Description of the indicators of absolute poverty

Indicator	Description
Material deprivation rate	Inability to afford some items considered by most people to be desirable or even necessary to lead an adequate life. It is defined as the proportion of people lacking at least three out of nine items describing these consumption goods and activities that are typical in a society, irrespective of people’s preferences with respect to these items (Törmälehto and Sauli 2010).
Material deprivation depth	Unweighted mean number of items lacked by the deprived population (Eurostat 2010).
Percentage of people experiencing difficulty in making ends meet	Percentage of people who have experienced difficulty or great difficulty in making ends meet.
Percentage of people experiencing problems with their dwelling	Computed by combining two EU-SILC variables: 1. the variable indicating the presence of leaking roof, damp walls/floors/foundation, rot windows or floor; and 2. the variable indicating other problems with the dwelling like not enough light.
Percentage of people living in over-crowded houses	Computed as the number of co-residents per room, i.e. crowding index. The crowding index is computed using the EUSILC variables household size and number of rooms available to the household. A threshold value of two is chosen to define crowded houses (^a^).
Percentage of people who cannot afford necessary medical treatments	Computed by combining two questions from the EU-SILC in order to describe situations when medical needs are unmet due to economic reasons only.
Percentage of people who cannot afford necessary dental treatments	Computed by combining two questions from the EU-SILC in order to describe situations when dental needs are unmet due to economic reasons only.

^a^Some previous analyses show that values of the crowding index higher than 2 are associated to critically low socioeconomic status (Melki et al. 2004)

### Relative Poverty

Relative Poverty component includes the three well -known Foster-Greer-Thorbecke (FGT) measures: poverty incidence *P*_0_, poverty depth *P*_1_ and poverty severity *P*_2_ (Foster et al. 1984, 2010) calculated according to the general formula:

1 $$ {P}_a\left(y,z\right)=\frac{1}{n}\sum_{i=1}^q{\left(\frac{z-{y}_i}{z}\right)}^a $$

where *α* is a real positive, *y* = (*y*_1_, *y*_2_, …, *y*_*n*_) is a vector of properly defined income in increasing order, *z* > 0 is a predefined poverty line, *n* is the total number of individuals under analysis, (*z*–*y*_*i*_)/z is the normalised income gap of individual *i* and *q* is the number of individuals having income not greater than the poverty line *z*. The parameter *α* can be seen as a parameter of ‘poverty aversion’: the higher *α*, the higher the relevance assigned to the poorest poor (Foster et al. 2010).

*P*_0_, *P*_1_ and *P*_2_ are computed for all EU regions using national poverty lines (defined as 60 % of the median national disposable income) and individual disposable income adjusted for cost of living, as described shortly below. The national, instead of the regional, disposable income is used to compute poverty lines in order to highlight the differences between regions within the same country, as suggested by (Betti et al. 2012).

Specifically, individual disposable income adjusted for housing costs is computed as follows:

$$ adjusted\ equivalised\ disposable\ income=\frac{\left( HY020- HH070\cdot 12\right)\cdot HY025}{HX050} $$

where:

- HY020 is the total household disposable income; in the EU-SILC it represents a comparable measure of household income across the EU^7^;
- HH070 is monthly total housing costs; they comprise structural insurance, services and charges (sewage removal, refuse removal, etc.), taxes on dwelling, regular maintenance and repairs, cost of utilities (water, gas, electricity and heating), mortgage interest payments for owners, rent payments for tenants, housing benefits for households whose house is rented for free;
- HY025 is a within-household non-response inflation factor used to correct for non-response distortions;
- HX050 is the equivalised household size according to the modified OECD approach:
  $$ HX050=1+0.5\cdot \left(H{M}_{14+}-1\right)+0.3\cdot H{M}_{13-} $$
  where *HM*_14+_ is the number of household members aged 14 and over and *HM*_13−_ is the number of members aged 13 or less.

Following suggestions by Eurostat, housing costs are deducted from both the individual disposable income and the poverty line, so as not to weaken too much the link between poverty and low living standards.

### Earnings and Incomes

The Earnings and Incomes component describes the monetary aspects of standards of living with three indicators: compensation of employees, net adjustable household income and median regional income. Compensation of employees captures the working conditions in the region, in terms of salaries, while the net adjusted household income provides the income corrected for the cost of services financed or subsidised by the government. Without this type of adjustment, household income is generally underestimated in countries with extensive public services, like in the Nordic member states, and overestimated in those where households have to pay for most of these services (EC 2010). The median regional income is computed from the equivalised household disposable income after correcting for housing costs. Detailed definitions of the indicators in this component are provided in Table 2. The choice of the median instead of the mean in the computation of regional average incomes is driven by the fact that, as the distribution of income is skewed, ‘… median consumption (income, wealth) provides a better measure of what is happening to the “typical” individual or household than average consumption (income or wealth) …’ (Stiglitz et al. (2009, pp. 13–14)).

**Table 2:** Description of the indicators of earnings and incomes

Indicator	Description
Compensation of employees	It refers to gross wages, salaries and other benefits earned by individuals in economies other than those in which they are resident, for work performed and paid for by residents of those economies. Compensation of employees includes salaries paid to seasonal and other short-term workers (less than 1 year), to the employees of embassies and of other territorial enclaves that are not considered part of the national economy and to cross-border workers.
Net adjustable household income	It is household disposable income that is adjusted for social transfers in kind. Social transfers in kind are goods and services such as education, healthcare and other public services that are provided by the government for free or below provision cost. It includes income from economic activity (wages and salaries; profits of self-employed business owners), property income (dividends, interests and rents), social benefits in cash (retirement pensions, unemployment benefits, family allowances, basic income support, etc.), and social transfers in kind (goods and services, such as healthcare, education and housing, received either free of charge or at reduced prices).
Median regional income	It is a median of equivalised household total disposable income after correcting for total housing costs.

SAS® ver. 9.2 was used for indicator extraction and computations.

## Aggregated Measures

The issue of aggregating indicators into a single, composite index is a widely debated topic in socioeconomics, especially when measuring poverty and quality of life (Decancq and Lugo 2013; Lustig 2011; Ravallion 2011; Wagle 2008). The aggregation process always implies, explicitly or implicitly, the choice of weights to be assigned to different, suitably selected and scaled indicators and the aggregation method. Both issues play a crucial role in determining the trade-offs between the different aspects measured (OECD-JRC 2008). Although we are aware that multi-criteria methods are analytical instruments to study these kinds of problems, like the counting method proposed by Alkire and Foster (2011a) or the purely multi-criteria approaches based on partial order (Annoni 2007; Annoni and Bruggemann 2009; Bruggemann and Carlsen 2012), within each poverty component we opt for a classical aggregation technique, as we assume, test and confirm an internal consistency of each component.

Indicators are then aggregated only within each poverty component. For all the regions in the analysis three separate aggregated measures are computed: Absolute Poverty Index (API), Relative Poverty Index (RPI) and Earnings and Incomes Index (EII). Following recommendations by different scholars on the topic, see for example Ravallion (2011) and Stiglitz et al. (2009), no aggregation is performed across the three components. They indeed describe different, and sometimes contradicting, aspects of people’s standards of living, which implies that it would make little sense to provide a single, aggregated measure of the three. Within each component we: (*i*) check for statistical internal consistency; (*ii*) standardise indicators by means of weighted z-scores; (*iii*) adopt an inequality-adverse type of aggregation; and (*iv*) use equal weights.

Principal Component Analysis (PCA) (Morrison 2005) is employed for internal consistency assessment. The aim is to check to what extent indicators within the same component measure the same latent variable. Internal consistency, which is related to the level of correlation or association among indicators, if established, reduces the effect of different weighting scheme on the final, aggregated measure (Decancq and Lugo 2013; Hagerty and Land 2007; Michalos 2011). In our case, selected indicators show a good level of internal consistency for all three components (Table 3 summarises the PCA outcomes). It can be seen that the share of variance explained by the first principal component (PC) is always very high. It varies from 74 % for Absolute Poverty to 95 % for Relative Poverty, suggesting that the indicators included are indeed measuring a single latent phenomenon in each of the three components. The analysis of the loadings, which are always statistically significant, shows that almost all the indicators contribute to the first PC to the same extent, supporting our choice of equal weights. The only exception is the ‘share of people living in crowded houses’ indicator that shows the lowest value among the Absolute Poverty indicators, namely 0.29, whereas all other indicators have a loading value higher than 0.37.

**Table 3:** Principal component analysis outcomes for each of three poverty components

Component	Number of indicators included	Variance explained by first PC	First PC loadings	
			Minimum value (corresponding indicator)	Maximum value (corresponding indicator)
Absolute poverty	7	74 %	0.29 (share of people in crowded houses)	0.42 (deprivation rate)
Relative poverty	3	95 %	0.57 (poverty rate)	0.59 (poverty depth)
Earnings & incomes	3	81 %	0.50 (employees’ compensation)	0.62 (net adjusted household income)

According to the well-known principle, particularly true when speaking of well-being, stating that deficiency in one element leads to a general failure, good living standards are ensured if all poverty indicators are at satisfactory levels. It implies, in turn, that shortages in one indicator of the poverty component cannot be fully compensated with surpluses in another indicator. In the aggregation procedure, full compensability can be avoided with generalised weighted means; this is supported in the literature of multidimensional poverty and inequality (Decancq and Lugo 2013; Ruiz 2011).

Let *x*_*ij*_ denote the value of indicator *j* (*j* = 1,…,*q*) for region *i* (*i* = 1,…,*n*). For each region the vector *x* = (*x*_1_,…,*x*_*q*_) is assumed available at a certain time point with the same positive orientation with respect to the latent phenomenon under analysis. A generalised mean of order *β* is defined as:

2 $$ I\left(x,\beta, w\right){\left[\frac{w_1f{\left({x}_1\right)}^{\beta }+\cdots +{w}_qf{\left({x}_q\right)}^{\beta }}{w_1+\cdots +{w}_q}\right]}^{\raisebox{1ex}{$1$}\!\left/ \!\raisebox{-1ex}{$\beta $}\right.}\beta \ne 1 $$

$$ I\left(x,0,w\right)=f{\left({x}_1\right)}^{w_1}\cdot \dots \cdot f{\left({x}_q\right)}^{w_q}\left( geometric\; mean\right) $$

where *f*(*x*_*j*_) represents transformed (standardised) indicators, and the vector *w* = (*w*_1_,…,*w*_*q*_) contains the indicator weights, such that *w*_1_+ … + *w*_*q*_ = 1. Our approach is based on the assumption of 0 < *β* < 1. Under this assumption the generalised mean is said to be inequality-adverse: a rise in the level of one indicator in the lower tail of the distribution will increase the overall mean by more than a similar rise in the upper tail, thus giving more importance to low levels (Ruiz 2011).

Generalised means of the type (2) satisfy a series of mathematical properties required for aggregated measures, especially in the field of welfare and inequality (Ruiz 2011). In our case we are particularly interested in the marginal substitution rate between indicator *j* and *k*—*MSR*_*j*,*k*_—which is defined as:

3 $$ MS{R}_{j,k}=-\frac{d{x}_j}{d{x}_k} $$

In case of aggregation of type (2), *MSR*_*j*,*k*_ depends on three elements:

1. weight dependency:
  4 $$ MS{R}_{j,k}\alpha \frac{w_k}{w_j} $$
2. transformation dependency:level dependency:
  5 $$ MS{R}_{j,k}\alpha \frac{f^{\prime}\left({x}_k\right)}{f^{\prime }x\left({x}_j\right)} $$
  where *f*’ indicates the first derivative of function *f*
3. level dependency:
  6 $$ MS{R}_{j,k}\alpha {\left[\frac{f\left({x}_j\right)}{f\left({x}_k\right)}\right]}^{1-\beta } $$

Weight dependency is generally recognised and corresponds to the role of the weights when performing linear aggregations. Transformation dependency is more subtle and not always clear to interpret. It influences the role of the indicators in a composite measure. For example, if we choose z-score standardisation, as in our case, the transformation-related element of *MSR*_*j*,*k*_ is the ratio of standard deviations *σ*_*j*_/*σ*_*k*_ of original indicators. The level dependency links different indicator levels (values) with the order *β* of the mean. The order *β* has the role of balancing the achievements between the two indicators *j* and *k*. Given that the indicator orientation is positive (the higher, the better), when *β* increases, more importance is given to the upper tail of the indicator distribution; while as *β* decreases, greater weight is given to the lower tail.

The generalised mean of power *β* = 0.5 is adopted. However an influence of different values of *β* in the interval [0,1] (from geometric to arithmetic mean) on final scores and ranks is tested through a Monte-Carlo exercise for each poverty component (Annoni et al. 2012; Saisana et al. 2005). The analysis shows only very minor differences in region scores and ranks, as expected given the high internal consistency of the indicators within each component (Decancq and Lugo 2013; Hagerty and Land 2007; Michalos 2011).

## The Distribution of Poverty Across EU Regions

### Absolute Poverty Index

**Fig. 2:**
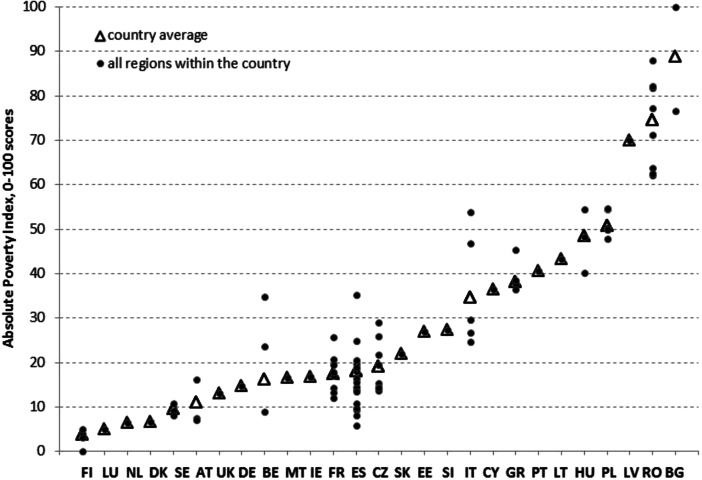
API scores—countries reordered according to the weighted country average (an explanation of the country codes is provided in Table 6. in the Appendix)

Figure 2 shows API scores for regions within each country. The countries are ordered from the best (low poverty levels) to the worst (high poverty levels), according to the weighted country average. The best countries, with the lowest levels of absolute poverty, are the EU Scandinavian countries (Finland, Denmark and Sweden), Luxembourg and the Netherlands. Central and eastern European (CEE) countries, Hungary, Poland, Latvia, Romania and Bulgaria, are the worst performing ones, with the last three characterised by an especially inferior situation regarding absolute poverty. In terms of within-country variability, which could not be measured for all the countries due to the limitation of data availability, Spanish, Italian, Romanian and Bulgarian regions are those showing the highest levels of variability (read inequality), while Swedish, Finish, Polish and Greek regions show the lowest.

Table 7. in the Appendix lists all the regions sorted from the lowest to the highest API scores (normalised from 0 to 100). The best 20 % of the regions,^8^ corresponding to the low levels of absolute poverty, include all Finnish and Swedish regions, two out of three Austrian regions (AT2 and AT3),^9^ the Netherlands, Denmark, Luxembourg, one Belgian region (BE2) and a few regions in the northern part of Spain (ES13, from ES21 to ES24). The worst 20 % of regions are almost all from the CEE countries, namely all Romanian and Bulgarian regions, five out of six Polish regions, Latvia and one Hungarian region (HU3). The only exception is insular Italy comprising Sardinia and Sicily (ITG).

### Relative Poverty Index

**Fig. 3:**
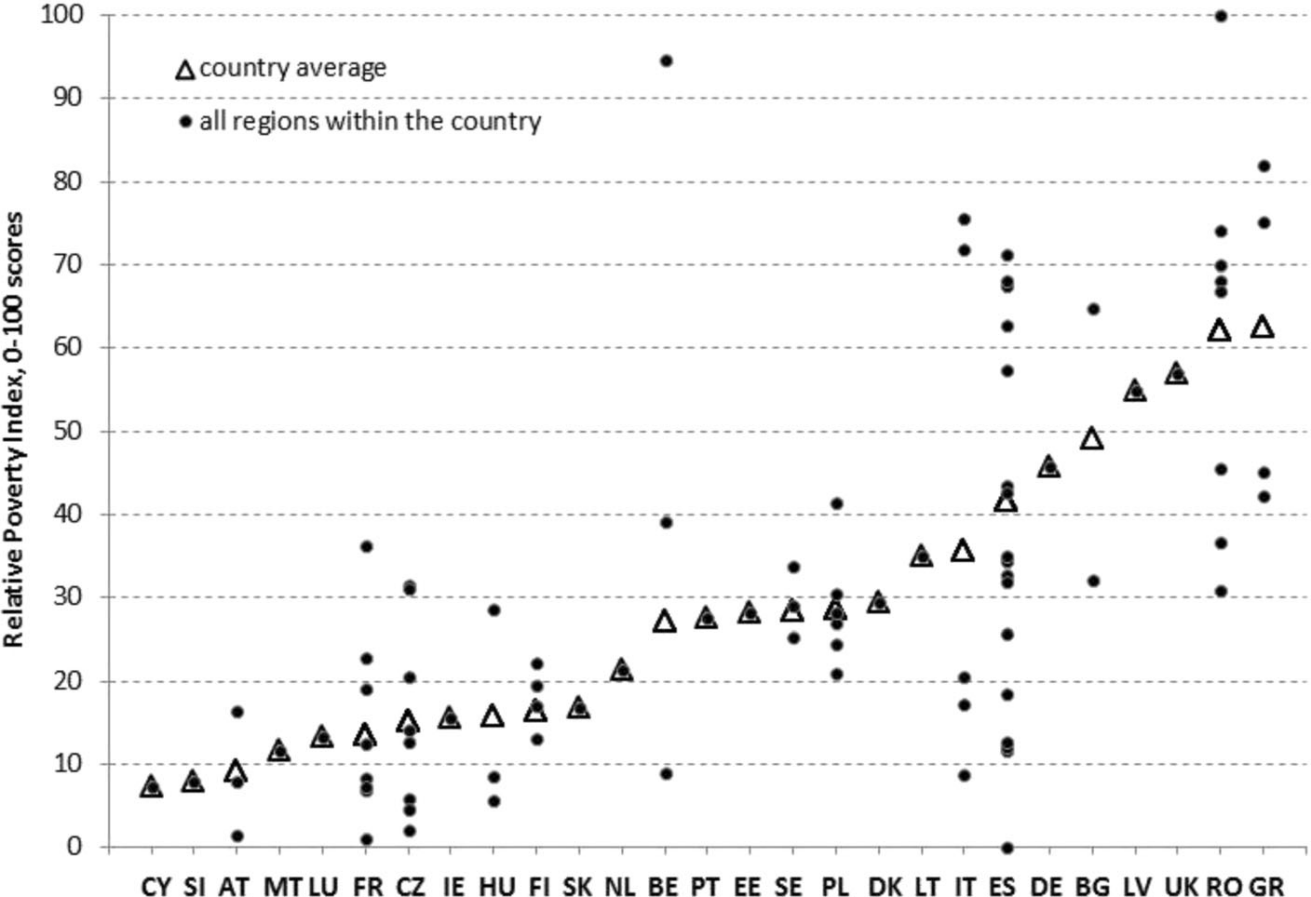
RPI scores—countries reordered according to the weighted country average (an explanation of the country codes is provided in Table 6. in the Appendix)

The poverty picture resulting from RPI scores changes considerably with respect to the one derived from API scores, confirming the intrinsic difference between absolute and relative measures of poverty (RPI scores are presented in Fig. 3). In this case, the lowest levels of relative poverty are observed in two southern European countries, namely in Cyprus and Malta, in one CEE country, namely Slovenia, and in Austria and Luxembourg. At the other end of the scale are three CEE countries—Bulgaria, Latvia and Romania—but also one southern European country, namely Greece, and the United Kingdom.

With respect to the RPI, the importance of sub-national analysis in measuring poverty is easily noticeable. It can be noted that the same country may comprise regions belonging to the top and bottom performers. The most striking examples are regions in Belgium, Spain and Italy in which, even with all the needed precautions due to regional data limitations, within-country variability of the RPI is extremely high.

RPI scores are shown in Table 8. in the Appendix, with regions reordered from best to worst. The best regions (with the scores lower than the P20 percentile) include four French regions (FR20, FR40, FR50 and FR70), three Czech regions (CZ01, CZ02 and CH03), two Hungarian regions (HU1 and HU2), two Austrian regions (AT2 and AT3), two Spanish regions (ES12 and ES22), one Italian region (ITD), one Belgian region (BE2), Cyprus, Slovenia and Malta. Among the worst performers (scores of the RPI above P80) are Latvia, the United Kingdom, five out of eight Romanian regions, two Greek regions (GR1 and GR2), two southern Italian regions (ITG and ITF) and one Bulgarian region (BG3).

### Earnings and Incomes Index

**Fig. 4:**
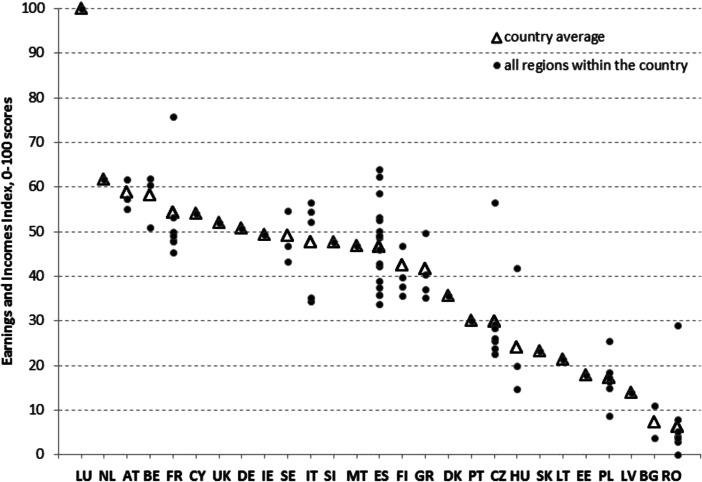
EII scores—countries reordered according to the weighted country average (an explanation of the country codes is provided in Table 6. in the Appendix)

In terms of EII scores (Fig. 4) the highest overall income and earnings values are decisively in Luxembourg, which is followed by the Netherlands and Austria. Then, slightly lower performance characterises the group of Belgium, France, Cyprus, the United Kingdom and Germany. The lowest overall income and earnings values are in the CEE countries, such as Estonia, Poland, Latvia, Bulgaria and Romania. Also in this case the sub-national variability, when measured, is relevant, especially in France, Italy, Spain, the Czech Republic, Hungary and Romania, highlighting the presence of high levels of inequality.

Table 9. in the Appendix lists all the regions reordered according to EII scores. The group of most affluent regions includes Luxembourg, the Netherlands, Cyprus, all Austrian regions, two French regions (FR10 and FR70), two Belgian regions (BE1 and BE2), three Spanish regions (ES21, ES22 and ES30), two northern Italian regions (ITC and ITD), one Czech region (CZ01) and the Swedish capital region (SE1). At the bottom end of the distribution, where scores of the EII are below the P20 percentile, one can find almost all Romanian regions (apart from the capital region RO32), two Bulgarian regions (BG3 and BG4), Latvia, Estonia, five out of six Polish regions (apart from the capital region PL1) and two Hungarian regions (HU2 and HU3).

### Shall we Aggregate Further?

The three poverty measures describe the concept of poverty from considerably different perspectives. Two of them, Absolute Poverty and Income and Earnings, are absolute measures of economic deprivation: the former in terms of non-financial household-related aspects, the latter in terms of income-related levels. Relative poverty is instead intrinsically different. It is indeed by construction a ‘relative’ concept, which basically captures the level of deprivation people experienced compared to those living in the same area. Low values of relative poverty do not necessarily imply that people are well-off; it shows a low level of heterogeneity of poverty across the population.

Our statistical analysis supports this reasoning. Table 4 shows that the three indices are interrelated both in terms of classical Pearson’s correlation (left side of the table) and rank correlation (right side of the table). Correlation levels are always statistically significant even if the RPI shows the lowest values. PCA outcomes (Table 5) indicate the presence of a strong first latent dimension accounting for 72 % of total variance almost equally explained by the three indices, as can be seen from the loadings of the first PCA component. Still, there is a second component of not scant relevance that accounts for 21 % of variance. Additionally, it is mostly driven by the RPI (with the loading of 0.84) and also characterised by the negative loadings of the API (− 0.21) and the EII (− 0.50). It means that in this component the two indices (API and EII) definitely contrast the RPI.

**Table 4:** Correlation matrix for scores and ranks

	Scores			Ranks		
	Absolute poverty	Relative poverty	Earnings & incomes	Absolute poverty	Relative poverty	Earnings & incomes
Absolute poverty	1.00			1.00		
Relative poverty	0.54	1.00		0.51	1.00	
Earnings & incomes	0.75	0.42	1.00	0.68	0.44	1.00

**Table 5:** Principal component analysis outcomes for three poverty components

PCA on the poverty indices					
	Eigen value	Variance explained	PC loadings		
			Absolute poverty	Relative poverty	Earnings & Incomes
1st component	2.15	72 %	0.63	0.51	0.59
2nd component	0.62	21 %	−0.21	0.84	−0.50
3rd component	0.23	7 %	−0.75	0.19	0.63

This can be interpreted as follows. On the one hand, there are some regions in the EU with pockets of poverty implying that a part of the population is classified as poor both in absolute terms and compared to other people in the region. These situations positively contribute to the correlation level between absolute and relative measures of poverty. On the other hand, there are regions where poverty is homogeneously spread and people are classified as poor in absolute terms but not in relative ones (in regions in which most of the population is worse off, relative poverty cannot be high by definition).

What is the worst case between the two? It is not up to us to decide. As our aim is to detect such a situation, we must mention that the detection is biased if further aggregation is carried out as it would level-off contrasting conditions. This is the main reason for not aggregating further in this case.

**Fig. 5:**
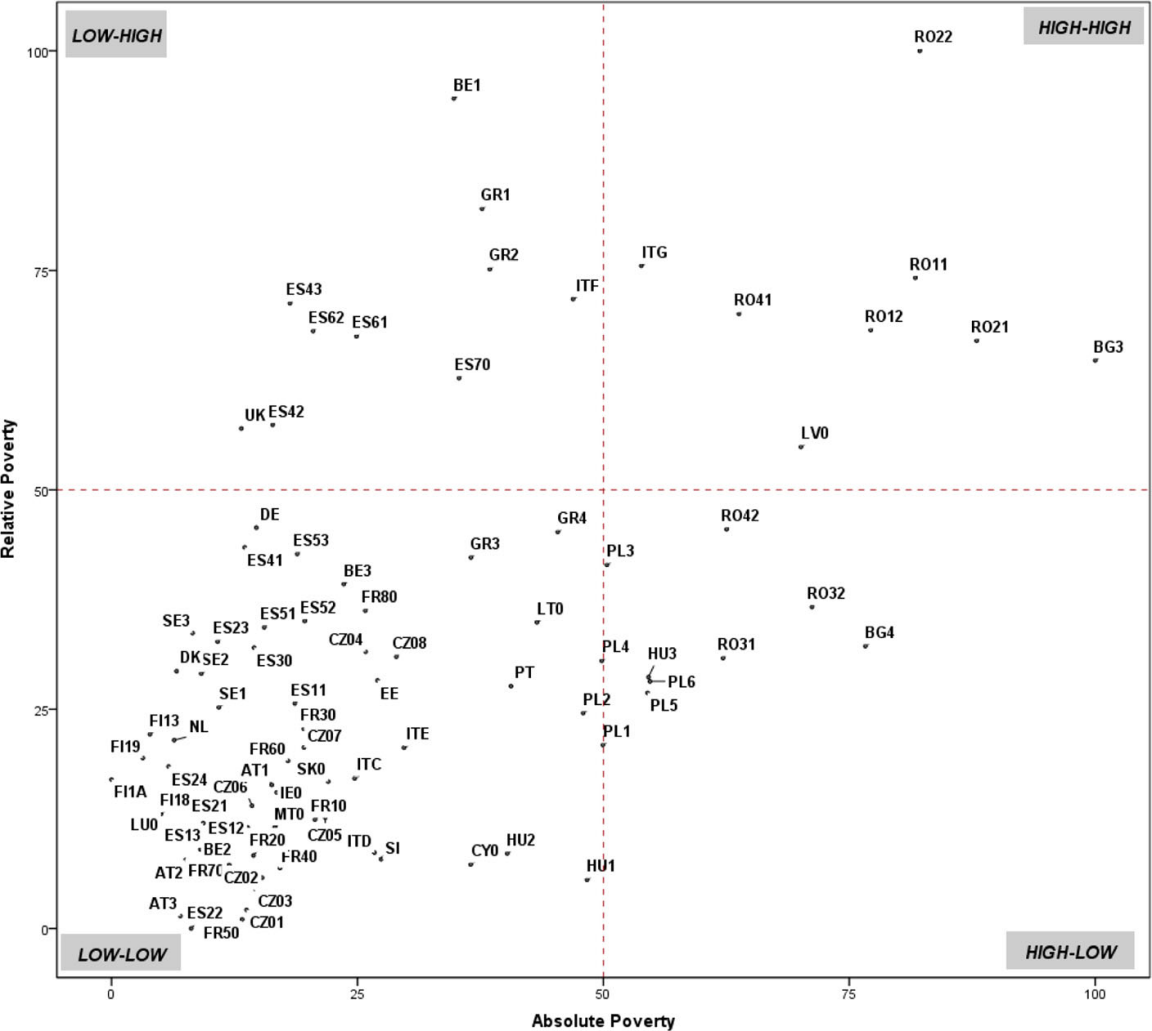
Correspondence between Relative and Absolute Poverty Indices (an explanation of the region codes is provided in Table 6. in the Appendix)

Table 10. in the Appendix provides separate regional rankings for the three indices. Among the three components of poverty, especially the concepts of absolute and relative poverty are substantially different and sometimes even in conflict. The scatterplot in Fig. 5 compares the API with the RPI. The scatterplot is divided into four quadrants—low-low, high-low, high-high and low-high, for an easier interpretation. Most of the regions are either in the low-low or in the high-high quadrant. It indicates that for these regions either low absolute poverty corresponds to low relative poverty meaning that overall people are well-off (bottom-left quadrant) or high absolute poverty corresponds to high relative poverty indicating a situation of deep and severe poverty (top-right quadrant). Some Romanian regions, Latvia, the northern part of Bulgaria (BG3) and the two biggest Italian islands—Sardinia and Sicily (ITG)—are in this serious poverty situation.

The top-left part of the plot comprises regions where, despite low absolute poverty levels, relative poverty can be deep and severe. As these regions may experience a high level of living standards inequality, this emphasises the presence of pockets of deprivation. This is the case of the United Kingdom and some regions in southern Europe, such as south-western Spanish regions (ES43, ES42 ES61, ES62 and ES70), the north-western regions in Greece (GR1 and GR2) and the most southern Italy (ITF), even if it is very close to the border of the quadrant. The contrary can be said for the regions in the bottom-right part of the scatterplot, which includes regions experiencing high material deprivation with rather low relative poverty. These are generally regions in the CEE countries, such as Bulgarian, Hungarian, Polish and Romanian regions. People living there are deprived but the deprivation is almost equally spread across the population.

## Summary

In the framework of the cohesion policy, the European Union (EU) provides funds to regions lagging behind with the aim of reducing poverty and social exclusion, among others. In this respect, there is a considerable need for measuring tools enabling better identification of regions both most in need and where investments are expected to have the highest impact. In this study, we measure poverty, understood as economic well-being, across the EU at the sub-national level. The proposed conceptualisation of poverty comprises three components for which aggregated measures are computed: Absolute Poverty Index, Relative Poverty Index and Earnings and Incomes Index. These indices evaluate poverty in absolute and in relative terms, taking into account monetary and non-monetary indicators by means of objective and self-assessed measures.

Our core data set is the main EU data source on living conditions and income, the EU-SILC, waves 2007–2008–2009. Because the EU-SILC is designed to be representative only at the country level, going regional is quite a challenge. Therefore, the appropriateness of regional analysis is statistically checked. Results suggest that for most regions the level of sub-national representativeness is acceptable. Yet, specific actions are taken to correct discrepancies in some cases. Eventually, poverty is assessed for a total of 88 EU regions using 13 indicators. This does not mean that we are not aware of the shortcomings and limitations of this approach. On the contrary, we consider our analysis as an exercise, more than the final recipe, which should raise awareness on the importance of the availability of reliable regional data.

Apart from the sub-national level, our study features two novelties: the adjustment of disposable income for housing costs and the adoption of a generalised weighted mean to aggregate indicators within a component, to penalise inequality and mitigate compensability. No aggregation is, however, performed across the three poverty components that, being intrinsically different, provide sometimes very different pictures of regional poverty. In particular, the comparisons of absolute and relative poverty measures show that there are quite a few regions in which people are well-off in absolute terms but not in relative ones and vice-versa. This clearly shows the multidimensionality of the poverty concept and gives justification not to further aggregate the three poverty measures so as not to blur the actual picture. Multi-criteria analysis would help in this case and is indeed the approach adopted in an ongoing project on the same data. Preliminary results set a flag on particular regions for which the aggregation can hide important contrasting patterns across the poverty measures.

Poverty was also shown to be a local concept, with high levels of within-country variability. This implies that, to be effective, the EU needs more targeted local policies and monitoring.

We see some implications for future research. First, in-depth empirical research, for example employing individual level data and multi-level modelling, is needed to test the usefulness of the three indices of poverty. Second, the availability of the most recent 2012 EU-SILC wave, not yet released at the time when this paper was written, will allow us to repeat the analysis for the 2010–12 period and compare pre- versus post-crisis poverty levels. Third, estimating the poverty indices over time will enable monitoring regional policy effectiveness. Last, a multi-criteria analysis of the three indices by partial order tools would allow summarising the overall picture across the EU while preserving the intrinsically multidimensional nature of poverty.

## Appendix

**Table 6:** Region names and codes and lowest regional level available in EU-SILC, waves 2007–2009

Country	NUTS level	NUTS Name
AT	NUTS 1	AT1=Ostösterreich, AT2=Südösterreich, AT3=Westösterreich
BE	NUTS 1	BE1=Région de Bruxelles-Capitale/Brussels Hoofdstedelijk Gewest, BE2=Vlaams Gewest, BE3=Région Wallonne
BG	NUTS 1	BG3=Severna i Iztochna Bulgaria, BG4=Yugozapadna i Yuzhna Centralna Bulgaria
CY	NUTS 0	–
CZ	NUTS 2	CZ01=Praha, CZ02=Střední Čechy, CZ03=Jihozápad, CZ04=Severozápad, CZ05=Severovýchod, CZ06=Jihovýchod, CZ07=Střední Morava, CZ08=Moravskoslezsko
DE	NUTS 0	–
DK	NUTS 0	–
EE	NUTS 0	–
ES	NUTS 2	ES11=Galicia, ES12=Principado de Asturias, ES13=Cantabria, ES21=País Vasco, ES22=Comunidad Foral de NavarraES23=La Rioja, ES24=Aragón, ES30=Comunidad de Madrid, ES41=Castilla y León, ES42=Castilla-La Mancha, ES43=Extremadura, ES51=Cataluña, ES52=Comunidad Valenciana, ES53=Illes Balears, ES61=Andalucía, ES62=Región de Murcia, ES70=Canarias
FI	NUTS 2	FI13=Itä-Suomi, FI18=Etelä-Suomi, FI19=Länsi-Suomi, FI1A=Pohjois-Suomi
FR	NUTS 1	FR1=Île de France, FR2=Bassin Parisien, FR3=Nord-Pas-de-Calais, FR4=Est, FR5=Ouest, FR6=Sud-Ouest, FR7=Centre-Est, FR8=Méditerranée
GR	NUTS 1	GR1=Voreia Ellada, GR2=Kentriki Ellada, GR3=Attiki, GR4=Nisia Aigaiou, Kriti
IE	NUTS 0	–
HU	NUTS 1	HU1=Közép-Magyarország, HU2=Dunántúl, HU3=Alföld És Észak
IT	NUTS 1	ITC=Nord-Ovest, ITD=Nord-Est, ITE=Centro (I), ITF=Sud, ITG=Isole
LT	NUTS 0	–
LU	NUTS 0	–
LV	NUTS 0	–
MT	NUTS 0	–
NL	NUTS 0	–
PL	NUTS 1	PL1=Region Centralny, PL2=Region Południowy, PL3=Region Wschodni, PL4=Region Północno-Zachodni, PL5=Region Południowo-Zachodni, PL6=Region Północny
PT	NUTS 0	–
RO	NUTS 2	RO11=Nord-Vest, RO12=Centru, RO21=Nord-Est, RO22=Sud-Est, RO31=Sud-Muntenia, RO32=Bucureşti-Ilfov, RO41=Sud-Vest Oltenia, RO42=Vest
SE	NUTS 1	SE1=Östra Sverige, SE2=Södra Sverige, SE3=Norra Sverige
SI	NUTS 0	–
SK	NUTS 0	–
UK	NUTS 0	–

**Table 7:** Absolute poverty index

Region	Absolute poverty norm score	Region	Absolute poverty norm score	Region	Absolute poverty norm score	Region	Absolute poverty norm score
FI1A	0	CZ01	13.725	FR1	20.670	GR4	45.365
FI19	3.168	ES12	13.835	CZ05	21.719	ITF	46.920
FI13	3.899	CZ06	14.272	SK	22.017	PL2	47.955
FI18	5.017	FR2	14.416	BE3	23.607	HU1	48.341
LU	5.030	ES30	14.497	ITC	24.706	PL4	49.827
ES24	5.790	CZ03	14.575	ES61	24.895	PL1	49.939
NL	6.373	DE	14.716	FR80	25.792	PL3	50.358
DK	6.608	CZ02	15.295	CZ04	25.832	ITG	53.852
AT3	6.996	ES51	15.525	ITD	26.726	PL5	54.504
AT2	7.513	AT1	16.277	EE	27.030	HU3	54.578
ES22	8.093	ES42	16.375	SI	27.401	PL6	54.744
SE3	8.221	MT	16.599	CZ08	28.945	RO31	62.169
BE2	9.034	IE	16.795	ITE	29.726	RO42	62.534
SE2	9.125	FR4	17.117	BE1	34.797	RO41	63.778
ES13	9.317	FR6	17.931	ES70	35.318	LV	70.080
ES21	9.761	ES43	18.143	CY	36.511	RO32	71.207
ES23	10.776	ES11	18.629	GR3	36.533	BG4	76.645
SE1	10.901	ES53	18.871	GR1	37.660	RO12	77.172
FR7	11.953	FR3	19.553	GR2	38.450	RO11	81.709
UK	13.191	CZ07	19.573	HU2	40.222	RO22	82.174
FR5	13.267	ES52	19.639	PT	40.612	RO21	87.950
ES41	13.544	ES62	20.493	LT	43.239	BG3	100.000

**Table 8:** Relative poverty index

Region	Relative poverty norm score	Region	Relative poverty norm score	Region	Relative poverty norm score	Region	Relative poverty norm score
ES22	0.000	FI18	13.084	PL6	28.180	ES41	43.416
FR5	1.038	LU	13.358	EE	28.284	GR4	45.213
AT3	1.440	CZ06	14.032	HU3	28.663	RO42	45.509
CZ01	2.133	IE	15.541	SE2	29.060	DE	45.687
CZ03	4.539	AT1	16.402	DK	29.335	LV	54.907
HU1	5.561	SK	16.781	PL4	30.529	UK	56.973
CZ02	5.795	FI1A	17.009	RO31	30.838	ES42	57.393
FR4	6.912	ITC	17.125	CZ08	30.999	ES70	62.724
FR7	7.253	ES24	18.453	CZ04	31.568	BG3	64.735
CY	7.313	FR6	19.109	ES30	32.021	RO21	66.992
AT2	7.821	FI19	19.429	BG4	32.202	ES61	67.492
SI	7.922	ITE	20.586	ES23	32.714	ES62	68.092
FR2	8.344	CZ07	20.635	SE3	33.697	RO12	68.191
HU2	8.566	PL1	20.916	ES51	34.364	RO41	70.055
ITD	8.669	NL	21.469	LT	34.926	ES43	71.251
BE2	9.046	FI13	22.139	ES52	35.074	ITF	71.755
ES12	11.547	FR3	22.745	FR8	36.247	RO11	74.167
MT	11.579	PL2	24.555	RO32	36.684	GR2	75.150
ES13	12.026	SE1	25.221	BE3	39.253	ITG	75.532
FR1	12.436	ES11	25.663	PL3	41.454	GR1	82.008
CZ05	12.659	PL5	26.855	GR3	42.293	BE1	94.611
ES21	12.737	PT	27.628	ES53	42.682	RO22	100.000

**Table 9:** Earnings and incomes index

Region	Earnings and incomes norm score	Region	Earnings and incomes norm score	Region	Earnings and incomes norm score	Region	Earnings and incomes norm score
LU	100.000	DE	50.686	GR4	40.414	CZ07	23.831
FR1	75.848	ES12	50.219	FI19	39.721	SK	23.241
ES21	64.074	FR4	49.976	ES70	39.006	CZ04	22.662
ES22	62.305	FR6	49.898	FI1A	37.647	LT	21.328
BE2	62.019	GR3	49.777	ES42	37.558	HU2	19.828
NL	61.810	IE	49.306	GR1	37.067	PL2	18.365
AT1	61.672	ES53	49.173	ES61	35.921	EE	17.939
BE1	60.452	ES23	49.052	ES62	35.821	PL5	17.238
ES30	58.663	FR8	49.000	DK	35.667	PL4	16.391
AT3	57.413	ES13	48.698	FI13	35.557	PL6	14.915
ITC	56.492	FR2	48.041	GR2	35.314	HU3	14.807
CZ01	56.491	FR5	47.942	ITG	35.291	LV	13.821
AT2	55.167	SI	47.586	ITF	34.436	BG4	10.966
SE1	54.658	FI18	46.766	ES43	33.863	PL3	8.797
ITD	54.496	MT	46.750	PT	30.150	RO42	7.858
CY	54.111	SE2	46.739	CZ02	29.225	RO41	5.250
ES24	53.306	ES41	46.025	RO32	29.057	RO11	4.293
FR7	53.260	FR3	45.316	CZ06	28.319	RO12	3.904
ES51	52.697	SE3	43.295	CZ03	28.306	RO31	3.787
ITE	52.177	ES52	42.817	CZ08	26.204	BG3	3.733
UK	51.955	ES11	42.301	CZ05	25.569	RO22	3.033
BE3	50.906	HU1	41.785	PL1	25.558	RO21	0.000

**Table 10:** Poverty: regional rankings of API, RPI and EII

Region	API	RPI	EII	Region	API	RPI	EII
AT1	32	28	7	FR4	35	18	25
AT2	10	22	16	FR5	21	7	33
AT3	9	8	11	FR6	37	29	26
BE1	58	75	5	FR7	19	13	18
BE2	13	12	6	FR8	51	55	28
BE3	48	52	22	GR1	62	73	47
BG3	88	78	85	GR2	63	76	52
BG4	83	57	79	GR3	61	41	23
CY	60	31	19	GR4	67	62	44
CZ01	24	1	10	HU1	70	6	42
CZ02	30	3	59	HU2	64	10	71
CZ03	28	2	63	HU3	77	40	76
CZ04	52	36	69	IE0	34	35	31
CZ05	46	4	65	ITC	49	25	12
CZ06	27	5	62	ITD	53	15	17
CZ07	42	14	67	ITE	57	46	24
CZ08	56	33	64	ITF	68	85	57
DE	25	51	15	ITG	74	87	56
DK	8	32	48	LT	66	67	70
EE	54	64	73	LU	4	23	1
ES11	39	69	45	LV	81	72	78
ES12	23	47	27	MT	36	30	39
ES13	15	48	34	NL	7	21	3
ES21	16	38	4	PL1	72	39	66
ES22	11	11	8	PL2	69	42	72
ES23	18	60	30	PL3	73	70	80
ES24	6	50	21	PL4	71	54	75
ES30	29	44	9	PL5	75	49	74
ES41	22	71	40	PL6	76	56	77
ES42	33	77	51	PT	65	59	60
ES43	38	86	58	RO11	86	82	83
ES51	31	53	20	RO12	84	79	84
ES52	43	65	43	RO21	87	80	88
ES53	40	58	29	RO22	85	88	87
ES61	50	84	54	RO31	78	61	86
ES62	45	83	55	RO32	82	68	61
ES70	59	81	49	RO41	80	74	82
FI13	3	37	53	RO42	79	66	81
FI18	5	9	36	SE1	17	26	13
FI19	2	27	46	SE2	14	34	35
FI1A	1	24	50	SE3	12	45	41
FR1	44	16	2	SI	55	17	37
FR2	26	19	32	SK	47	20	68
FR3	41	43	38	UK	20	63	14

^a^Low ranks indicate low poverty and high ranks indicate high poverty

**Table 11:** Abbreviations and meanings

Abbreviation	Label
API	Absolute poverty index
AROPE	At risk of poverty or social exclusion
CEE	Central and Eastern European
CP	Cohesion policy
EII	Earnings and incomes index
EU	European union
EU-SILC	European union statistics on income and living conditions
FGT	Foster-Greer-Thorbecke
GDP	Gross domestic product
NUTS	Nomenclature of territorial units for statistics
OECD	Organisation for economic co-operation and development
PCA	Principal component analysis
RPI	Relative poverty index

## Footnotes

1
http://ec.europa.eu/europe2020/targets/eu-targets/index_en.htm

2 Nomenclature of territorial units for statistics (NUTS) is a hierarchical system for dividing up the economic territory of the EU. NUTS 0 level corresponds to the national level, while NUTS 1, NUTS 2 and NUTS 3 correspond to the sub-national levels: the higher the level, the smaller the territorial unit.

3 An alternative proxy for the costs of living is the imputed rent (Frick et al. 2010). Because estimation of imputed rent is still very problematic (Törmälehto and Sauli 2010), this option is not considered.

4 We are aware that by averaging across 2007, 2008 and 2009 waves we provide a snapshot of poverty in the EU just before the start in 2008 of the financial and then economic crisis. The crisis has very likely worsened the picture presented here. With the availability of micro-data for the 2012 wave, expected in 2014, we plan to repeat the analysis with the three most recent waves, namely 2010–2012. We recall that the EU-SILC income data refer to the total annual income of households in the year prior to the survey with the exception of the United Kingdom (for which the household income is calculated on the basis of current income) and Ireland (where the calculation is based on a moving income reference period covering part of the year of the interview and part of the year prior to the survey) (Fusco et al. 2010).

5 Cyprus, Estonia, Lithuania, Luxembourg, Latvia and Malta are small countries with no administrative regions. Germany, Denmark, the Netherlands, Portugal, Slovakia and the United Kingdom do not provide regional identifiers making it impossible to disaggregate the indicators at the sub-national level.

6 Even if the poverty-related questions are present in country-specific household surveys, they are often not formulated in the same way with respect to their content, wording, answer categories, etc., thus hampering comparability.

7 Disposable income is the most common indicator of economic resources used in poverty studies (McNamara et al. 2006). The EU-SILC defines household disposable income as the sum of a number of household and personal income components (Eurostat 2010): (1) gross (or net) personal income components of all household members, like employee income, company car, profits or losses from self-employment, unemployment benefits and other benefits (+); (2) gross (or net) income components at household level, like income from rental of a property or land, family or housing -related allowances, interests or profit from capital investments, regular (+); (3) inter-household cash transfers received and other types of household incomes (+); and (4) deductions, like taxes on income, social insurance and wealth, inter-household cash transfer paid (−).

8 20 % of the highest scoring regions (corresponding to the P80 percentile) and 20 % of the lowest scoring regions (corresponding to the P20 percentile) are chosen because we make reference to the P80/P20 inequality measure.

9 Region names are provided in Table A.1. in the Appendix.
